# Extracts and Gleanings

**Published:** 1876-07

**Authors:** 


### Extracts and Gleanings.

*Post Nasal Catarrh.*—Dr. Robinson has given an extensive trial to all the medicaments which are supposed to have a special effect upon the mucous squill, copaiba, cubebs, iodide of potassium, ammoniacum, guaiac, etc., and has found three of especial benefit—sulphur, cubebs, and ammoniacum. He constantly uses the sulphur water of Sharon Springs, and believes that sulphur is much more beneficial given internally than as a local application. Of cubebs he formerly employs the oleo-resin, but has since found the powder much more efficient. The following is a good formula for its exhibition :

R—Pulv. Cubebæ, ozi.j ;
Syrupi Aurant. Cort., f. oz. iij. ;
Aq. Menth. Pip., f. oz. ij ;
Aquæ, f. oz. iij.

S. Tablespoonful every three hours.

It sometimes causes nausea, diarrhœa or a slight eruption on the skin, but these may be easily arrested by diminishing the dose. The use of the agent should be persevered in for a long time, but at intervals it should be discontinued for a few days. Ammoniacum may be advantageously combined with ipecacuanha and carbonate of ammonia, and is very serviceable in diminishing the secretion. When there is a constitutional taint of any kind suspected, the treatment must, of course, be modified to suit the circumstance. The patients suffering from malaria, fifteen to twenty grains of quinine should be given daily, gouty diathesis, guaiac in sufficient quantities. When syphilis is present, Dr. Robinson thinks a long continued and systematic course of mercury (preferable the bichloride and biniodide) is imperative, whatever may be the stage of the disease. * * * * He prefers the use of powders and sprays, which can be applied to as great an extent of the pituitary membrane as the douche, with no danger of causing otitis, while there is the positive advantage of being able to use them in concentrated form without occasioning the same annoyance to the patient. The spray should be applied at the temperature of the atmosphere the patient is accustomed to breathe, and should not be used in sufficient strength to do mischief. A useful combination is that of carbolic acid with borax in glycerin and water, which may be used with great benefit when ammoniacum is being given internally, as it prevents the masses of mucus from becoming hard and dry. One of the best applications in the form of a powder is a mixture of camphor and iodoform. The dry vapor of iodine, carbolic acid, and the essential oils can also be advantageously employed in some cases. Still, no local treatment can possibly restore the diseased follicles to their normal condition, as this can only be accomplished by persistent constitutional treatment, including a good hygienic regime.

Dr. L. D. Bulkley mentioned disorder of the liver as a frequent cause of nasal catarrh, and one to which Dr. Robinson had not alluded. His attention was first called to the subject in Murchison's lectures on Functional Diseases of the Liver. In his own person he had always been subject to the exact symptoms Dr. Robinson had described whenever he was bilious and his digestive powers impaired, and on investigation he had found this was true of a large number of his patients also. Many of those suffering from eczema, acne, and psoriasis, accompanied by lithæmia and derangement of the liver, were also affected with catarrh, which he had now learned to attribute to the same cause. In such cases he had found great benefit to be derived from the simple inhalation of steam impregnated with the compound tincture of benzoine. The method he recommended was to put a teaspoonful of the tincture (to which a little laudanum might be added, if there was much irritation of the fauces) in the bottom of a tin pan, and then pouring a pint of boiling water upon it, let the patient inhale the vapor. This simple and inexpensive method might be repeated as much as twenty times a day with great benefit.—*The Richmond and Louisville Medical Journal.*

*Salicylic Acid in Diphtheria, and in Rheumatism*—Quite a furor has arisen in some quarters, especially among German practitioners, on behalf of salicylic acid in diphtheria. It is employed as a gargle, or internally for young children who cannot use a gargle, or in both ways simultaneously. The gargle is made by dissolving one part of the acid in 100 parts of water, with the addition of a small quantity of alcohol. This solution is adapted also for internal use. The dose is from two to five grains, in powder or solution, every hour or two. According to one observer, the membrane begins to be cast off after the third or fourth dose. "Fifteen cases successfully treated"—"thirty-one cases without a single death"—"six cases with wonderful results"—so run the reports in favor. From other sources come counter reports. It is no better than chlorate of potash—says one. It is not equal to carbolic acid—says another. Like all new remedies, it never fails in the hands of a certain class of practitioners, whose imagination is superior to their judgment. Five or ten years hence salicylic acid will be almost forgotten. It will meet the fate of nineteen out of twenty new remedies—to sink below its real merits. Meanwhile it will be lauded as a cure for a variety of diseases. We have already been instructed as to its power to control fevers, and to cure intermittents. Its latest triumph is in acute rheumatism. Ten grains, more or less, must be given every hour until the diseased joints can be moved without pain. This will happen within forty-eight hours, and after taking from 75 to 250 grains. Professor Traube, of Berlin, is the authority for these statements. Perspiration, ringing in the ears and deafness are apt to ensue. One patient took upwards of five drachms in twelve hours, without disturbing his stomach. The medicine is acrid, and the mouth should be protected from contact with it. It passes rapidly into the urine, and can be detected there by chloride of iron, which produces a violet-blue reaction.

Dr. Da Costa, of Philadelphia, extols salicylic acid as an effectual remedy for foul breath. Instead of using alcohol to dissolve it, he adds borate of soda and glycerin—five grains of each, in half an ounce of water, with one drachm of glycerin.

This dose is given three times a day. The same solution may be employed as a gargle, or with the atomizer.—*Pacific Medical and Surgical Journal*, March, '76.

*Treatment of Placenta Prævia.*—Dr. T. Gaillard Thomas, after narrating to the New York Obstetrical Society (*American Journal of Obstetrics*), the notes of a case of placenta prævia, made the following remarks: Is it better to allow a pregnancy, during which the woman has become exsanguined and dangerously reduced by repeated hemorrhage from placenta prævia, to go on to term, or should premature labor be induced? He chooses the latter alternative, and has lost but one case of placenta prævia in which he brought on labor prematurely; the case died of post-partum hemorrhage. The children, of course, usually succumb. In the case just mentioned he detached the placenta (which was centrally inserted), cut the cord and removed it, leaving the child in the uterus; no hemorrhage occurred, and twenty-four hours later the child was safely expelled. The uterus contracted well apparently, but three hours afterwards the family physician was hurriedly called and found the lady dying of hemorrhage. In his opinion the induction of premature labor offers greater safety, both to the mother and the child, than the plan of allowing the pregnancy to go on to term. The hemorrhage from this malposition of the placenta generally occurs suddenly, often at night, and before the physician can reach the patient, she is beyond medical aid, or at least at the point of death. These repeated depletions also debilitate the child, and the question arises whether a child born prematurely at the eighth month is not fully as likely to live, or more so, than one weakened by repeated hemorrhage. If the labor is induced by rubber bags, the hemorrhage will be slight and the danger to the mother not great, for these rubber dilators compress so thoroughly as to arrest the bleeding from the placenta during the dilatation of the os; of course the diagnosis should be correct, and a granular endocervicitis producing occasional discharge of blood should not be mistaken for placenta prævia. This method of treatment is not mentioned in the obstetrical text-books.—*Canada Medical Record*, June, 1876.

*Treatment of Constipation and Diarrhœa with Water and Tincture of Camphor.*—Every one knows how difficult defecation sometimes is for those persons who are afflicted with habitual constipation. In such cases, relief is often sought from injections of cold water, with the occasional additions of oil or soap. The following clyster is recommended as infallible in such cases:

Take a tumbler and fill it half full of water at the temperature of the room, pour in a few drops of tincture of camphor, just enough to give the water a slight sapidity, then fill the glass with water. Inject this slowly into the rectum till about sixty or eighty grammes have been introduced. At first no effect is perceived, but in about ten minutes the desire to defecate becomes irresistible. The effect becomes energetic in proportion to the quantity of tincture of camphor added. After the defecation, it is well to repeat the injection of a small quantity of the same mixture and retain it in the rectum, which can readily be done, so as to prevent constipation on the following day.

Although at first sight it may seem rather improbable, the same remedy is also useful in checking diarrhœa, even though it may have been persistent. The injection should be extremely slow; after the dejection it should be repeated two or three times. The quantity of tincture of camphor should also be increased.—*New York Medical Journal.*

*Treatment of Cleft Palate without Operation.*—The *Lancet* states that in St. Thomas' Hospital Mr. Mason has, at the present time, under observation, several interesting cases of congenital cleft palate, which he is treating by the application of strong nitric acid alone, and consequently without the use of the knife. The ages of the patients vary from a few weeks to several years. Mr. Mason thinks that this method of effecting union is especially applicable to cases in which the cleft is of average extent, and even where the hard palate is partially implicated. In more severe instances the ordinary operation may be required. Mr. Mason finds that the application of the acid is attended with no pain or inconvenience whatever to the patient, and although the cure is more slowly accomplished, it has the advantage of being sure, and of completely closing the fissure in the most perfect manner, without the risk of the parts giving way, either wholly or partially, as too often happens after the usual operation of staphylorraphy. A further gain seems to be that the cases may be dealt with as out-patients, as in all the examples now under notice. Mr. Mason, after many trials, prefers the strong nitric acid to any other form of caustic.—*Med. and Surg. Rep.*

*On the Rational Treatment of Common Tapeworm.*—At a late meeting of the Medical Society of London, Dr. Brunton read a paper on this subject (*Lancet*, December 4, 1875). After stating the varieties met with in this country, and mentioning the anomalous symptoms to which they give rise (their very anomaly, he remarked, affording a clue to the diagnosis), he stated that the chief points to be observed in the treatment were: a preliminary starvation of twenty-four hours, and the administration of a combination of kameela and male fern—namely, two drachms of kameela to be rubbed up with a little gum and water till an emulsion is formed, and then two drachms of oil of male fern to be added, and the whole triturated in a mortar, with a gradual addition of water till a three-ounce mixture is formed, of which half is to be given at bed-time, and the remainder four hours later. This he had never known to fail. He insisted on the quality of the drugs being good, and spoke of the after-treatment by tonics.—*News and Library*, Jan., 1876.

*A Cure for Drunkenness.*—The American *Journal of Pharmacy* says that in Russia, for some time past, herba serpylli (wild thyme) has been used with great success to effect a permanent cure of drunkenness; in case of a relapse (only after years), a short treatment will effect a cure again. The treatment consists in making an infusion of wild thyme (1½ oz. to 1½ pints), and giving the patient a teacupful every half honr. The next day it is given every two hours, and then four to six times a day until the cure is complete, which generally takes from two to three weeks. The effects are in the following order: Vomiting, diarrhœa, increased urine, strong transpiration; then, generally, increased appetite and craving for acidulous beverages. The diet, easily digested food, and lemonade or other acidulous liquids.—*Medical and Surgical Reporter.*

*Iodoform.*—This therapeutic agent possesses, in a wonderful degree, the power of reducing inflammation, soothing pain, and rapidly healing excoriated and ulcerated surfaces, by whatever cause produced. As a topical application, its healing and anæsthetic powers are unequaled by those of any known agent. I am daily using it in the form of suppositories in cases of acute vaginitis with almost magical effect, and even in inflammation and induration of the neck of the womb it has proved itself an invaluable agent in rapidly relieving pain, reducing inflammation, and softening and restoring indurated tissue to a normal condition.

I am also successfully using iodoform, in ointment form (forty grs. to one ounce ung. simplex), in painful glandular enlargements. It has, in my own practice, shown itself as efficient as iodine. In connection with the topical application of this agent I daily administer three one-grain pills, at intervals of four hours each. For all such ills as these, iodoform has accomplished much already, and is destined, as I believe, to be of greater benefit, in the hands of the physician, than any known remedy. —*J. H. Johnson, M.D., in Boston Journal of Chemistry.*

*Employment of Aspiration in Intestinal Obstruction.*—We learn from the *Medical Times and Gazette* (London) that in a note laid before the Academie des Sciences, M. Demarquay states that the idea had occurred to him whether the same result that is obtained in gastro-enterotomy might not be attained by a simpler procedure, which might be employed by any surgeon. When an obstacle suddenly opposes the course of the contents of the intestine, gas accumulates above the obstruction, producing tympanitis, which is also accompanied by nausea and vomiting, the intestinal canal becoming paralyzed by the excessive distention. If, then, at the commencement of the affection, before any local or general peritonitis has supervened, we are able to relieve the tympanitis by an artificial removal of the gas, we find the intestinal movements are sometimes re-established, and with these a disappearance of the obstacle occurs. M. Demarquay has met with three cases in which he has had recourse to this procedure with success.—*Drug Circular.*

*Treatment of Severe Sprains.*—Mr. Sampson Gamgee gives a case of sprain of the ankle treated by compression, with very satisfactory results, and says: "Severe sprains are often serious fractures, though no bone be broken, or only a bit may be chipped off; the ligaments and fasciæ are ruptured, blood being extravasated into the joints, into the sheaths of tendons, and for some distance not infrequently between the layers of muscles. The swelling is great, the pain intense. The orthodox treatment by leeches and fomentations is valueless, compared with circular compression and perfect immobilization. Not only can the patient bear well-applied pressure from the first, however great the swelling and acute the pain, but it may be laid down as a general proposition, to which I have never seen an exception, that in severe sprains effusion is most surely checked, and, once it has occurred, its absorption is most rapidly promoted, while pain is most effectually relieved, by pressure and immobilization. It is as true now as when Velpeau taught it, that 'compression is the sovereign resolvent in contusions with infiltration and swelling.'"—*Lancet*, April 29, 1876.

*Dangers of Absorption of Carbolic Acid.*—The carelessness with which non-professional journals take up certain popular receipts is not always without its dangers, and especially since these often come into the hands of children. For instance, there appeared lately in one of the public prints an article upon the poison of vipers, which recommended that carbolic acid should immediately be introduced within the wound, the acid to be mixed with alcohol *in the proportion of two to one.* Observe the off-hand manner in which a toxic agent is spoken of, as if it were the most inoffensive thing in the world. In order to try the experiment, a cat was selected upon whose skin, denuded of hair alone, a saturated solution of carbolic acid in alchohol, with an equal quantity of water, was rubbed. This produced no effect; but when the same solution was rubbed into a scratch upon the nose two or three times, the animal immediately fell into convulsions, and very shortly succumbed. Prussic acid could not have acted more promptly. The moral of this experiment is obvious.—*Med. Times.*

*Quinetum.*—A preparation of the whole alkaloids separated from East India red bark, has been used for some time in the Indian hospitals, as well as in private practice, with great success. The concurrent testimony of medical men in our Indian possessions is to the effect that quinine is not so greatly superior to the whole alkaloids as to make it worth while to separate the sulphate in its pure state. Mr. Thomas Whiffen of the Quinine Works, Battersea, now offers to the profession a similar preparation, which he calls quinetum. It is in the form of a fine granular non-adherent powder of a pale buff color. The proportions of the various alkaloids present will of necessity vary with the sample of bark used; but, we think, not so much as to be of moment therapeutically. Sulphate of quinetum is a white crystalline body with a faint pink tinge, greatly resembling sulphate of quinine; and we are informed that the preparation can be supplied to the profession at about one-half of the cost of quinine.—*Brit. Med. Jour.*

*Ulcerative Stomatitis.*—It is now eight years since a physician, who had been fifty years in practice, suggested for a patient of mine, suffering from ulcerative stomatitis, a mixture of Peruvian bark, acacia, and sugar, made into a paste with cream; its success then led me to use it in one case after another, with the same result; and in scarlatina, diphtheria, and all aphthous affections, it has proved invaluable. The following is the formula: ℞—Cort. Peruv. flav., 2 dr.; acaciæ pulv., one dr.; sacch. alb., half dr. M. Sig. Mix one-half of this powder in a tablespoonful of cream, and apply frequently with a camel's hair brush.—Holden—*N. Y. Med. Jour.*, Dec., 1875.

*A New Method of Administering Enemata of Chloral.*—M. Dujardin-Beaumetz has employed the process suggested by Griffith for administering chloral enemata with success. A solution conraining a drachm of chloral is beaten up with the yolk of an egg, and this is added to a glass of milk to form the enema. This mixture has the advantage of causing no pain, which is not the case when doses of thirty to sixty grains of chloral are administered in the usual enema or suppository.—*Bull Gen. de Therap.*, June 15.

*Leptandra as a Purgatave.*—Dr. Wollen writes to the Louisville *Med. News:* "During the past few years I have been prescribing the *fluid extract of Leptandra,* with results so gratifying that I desire to call attention to the virtues of the above-named drug. Whether or not it has any specific action upon the liver I cannot say, but I am certain that it is one of the most efficient and safe purgatives that we have, and can be used without fear of debilitating the patient; in this respect widely differing from podophyllin, a remedy so often prescribed in hepatic disorders.

"In that class of cases usually called bilious, whatever that may mean, where there is loss of appetite, with a heavily-coated tongue and constipation of the bowels, I know of no single remedy universally applicable and useful as the leptandra. It is often well to combine it with the fluid extracts of senna and rhubarb; but whether so combined or given alone, it is certain in its results, and produces purgation without pain, griping or debility. In those cases of obstinate constipation occurring in young infants, where it is necessary to resort to a purgative, it will be found that a few drops of the leptandra added to the syrups of senna and rhubarb will produce the best results.

"It must be borne in mind, however, that the proper dose will vary with the individual. Owing to idiosyncrasy or other cause some patients will need but ten minims at a dose, while others will require as much as a drachm to produce any decided effect. The disagreeable taste of the drug is the only bar to its use, and this may be overcome to some extent by combining it with some syrup that is agreeable to the taste of the patient."—*Med. Brief.*

*Gonorrhœal Rheumatism.*—On this affection Dr. Geddings, in the Charleston *Medical Journal,* says: Adopting the view that gonorrhœal rheumatism originates in the absorption of noxious material from the inflamed or irritated urethral (in the male) and the urethro-vaginal (in the female) mucous membrane, every effort should be directed to the mitigation of the primary disease. This focus of infection must be removed before the chances of relieving the joint affection by general or local remedies can be considered as at all encouraging. The gonorrhœa or other urethral irritation must be actively treated. To this end, absolute rest, spare diet (in the robust) in cases of severe chordee or deep seated pain in the perinæum, leeches and mild purgatives are indicated. But, inasmuch as gonorrhœal rheumatism usually occurs after the acute symptoms of *clap* have subsided, we may in most cases proceed at once to the use of astringent injections, with a view to suspend the discharge. The following formula, suggested by Niemeyer, I have found very efficacious in simple gonorrhœa, and there is no reason why it may not prove equally so in complicated cases:

R. Acid tannic.................. .....grs. xxx—lx.
Vini Gallici.......................rub. f. oz. iv.
M. ft. inject. To be used three times a day.

*Psoriasis—Tar Internally.*—By Dr. T. M. C. Anderson.—Dr. A. states that he has come to regard tar in the light of one of the most valuable remedies we possess in the treatment of psoriasis. And it is not merely in mild cases that it does good, for in his hands it has frequently yielded the most satisfactory results in very obstinate cases after long courses of arsenic and many other orthdox remedies had been tried in vain. Dr. A. generally begins with two minims three times a day in a teaspoonful of treacle, and gradually increases the dose, if necessary, to half a teaspoonful, or even more. The small dose is advisable at first, as in some persons the medicine cannot be tolerated, and produces derangement of the digestive organs, fever and a bright red rash upon the skin. He testifies also to the virtues of this remedy in catarrh of the bronchial tubes, as pointed out by Dr. Ringer, and in chronic affections of the mucous membranes generally: he concludes with the remark "that it is very singular how such a valuable remedy, which seems in earlier days to have been highly esteemed, should, as an internal medicine, have fallen into disrepute in our time."—*Brit. Med. Jour.*, Apr. 24, 1875; Also, *The Am. Med. Jour.*, March, 1876.

*Poisoning by Santonin, and its Relief.*—P. Becker (*Centralblatt fur Med.*, reports the following case: A two-year old child was seized, two hours after taking 0. 1 grm. of santonin, with severe convulsions, beginning in the face, then the extremities, and finally interfering with respiration. The third and seventh pairs of nerves were the peculiar seat of the irritation. The pupils were enlarged. The urine showed the usual yellow color. Warm baths, enemata of vinegar, draughts of water, and artificial respiration were employed. The threatening paralysis at last diminished, and at the end of three days the child was well. The absence of any certain therapeutics led Becker to enter upon a series of experiments upon animals, with a view to discover some antidote to the poisonous effect of santonin.

Nitrite of amyl was employed, but without effect; morphia, by injection, likewise failed to avert the toxic result. Chloral hydrate, however, given before the administration of the santonin in quantity sufficient to cause sleep, prevented the occurrence of spasm.

Chloroform and ether by inhalation prevented the spasm, and animals thus treated recovered after the reception of otherwise fatal doses of santonin.

Artificial respiration, by means of tracheotomy and the bellows, acted less favorably than the above-mentioned hypnotics.

In cases of santonin-poisoning Becker recommends the inhalation of ether or chloroform, with artificial respiration during the first period, and, when the immediate danger is past, chloral carefully administered. Later, to remove the poison, laxatives, etc., may be employed.

*Prophylactic in Cholera Infantum.*—The author of this paper very justly states, that the various kinds of treatment adopted by physicians have not proved very successful in this malady; hence a prophylactic against it is of great value. As it originates in the nourishment of the infant, Jacusiel (Berlin *Klin. Wochenschrift*, 1875,) has been led to add two tablespoonfuls of one-third per cent. solution of salicylic acid in water to the daily allowance of milk, with the effect of rendering the germ of the disease powerless. The children fed in this manner have not had gastro-intestinal catarrh, or suffered any inconvenience from this rather free use of salicylic acid. The remedy is harmless and also inexpensive.—*Am. Med. Jour.*, March, 1876.

*The Abortive Treatment of Dysentery.*—Dr. David Prince (St. Louis *Med. and Surg. Journal,*) believing that in dysentery the indications for treatment are to produce a diarrhœa, to counteract or suppress the specific inflammation, and to secure rest to the alimentary canal, has made trial of chloral hydrate and sulphate of magnesium with very satisfactory results. As an abortive of dysentery, chloral is given in a dose large enough ordinarily to produce sleep, in connection with an ounce or two ounces of sulphate of magnesium, with sufficient water to make a solution. An average dose is thirty grains. As an irritable stomach often leads to the rejection of the medicine, it is always best to be prepared with a second dose to administer immediately, while the stomach is empty. A sinapism upon the scrobiculus cordis, or a cold wet napkin upon the neck, may aid in the retention of the dose. The subcutaneous injection of morphia, waiting a few minutes for its quieting effects, is an efficient aid in rendering the stomach tolerant of the dose.

In *The Clinic*, September 11, 1875, Dr. S. H. Lambert reports a very severe case of dysentery which he treated successfully with hydrate of chloral. He says that in similar cases nothing has given him more satisfaction than a full dose of fluid extract of aconite, followed by chloral.

*Treatment of Constipation and Diarrhœa.*—The New York *Med. Jour.*, quotes the *Gaz. Med. di Roma*: "Take a tumbler and fill it half full of water at the temperature of the room, pour in a few drops of tincture of camphor, just enough to give the water a slight sapidity, then fill the glass with water. Inject this slowly into the rectum till about sixty or eighty grammes have been introduced. At first, no effect is perceived, but in about ten minutes the desire to defecate becomes irresistible. The effect becomes energetic in proportion to the quantity of tincture of camphor added. After the defecation it is well to repeat the injection of a small quantity of the same mixture and retain it in the rectum, which can readily be done; so as to prevent constipation on the following day. Although at first sight it may be rather improbable, the same remedy is also useful in checking diarrhœa, even though it may have been persistent.

The injection should be extremely slow; after the dejection it should be repeated t·vo or three times. The quantity of tincture should also be increased.—*Med. and Surg. Reporter*, March 18, 1876.

*Treatment of Acute and Chronic Rheumatism by Means of Hot Packing.*—The method of procedure is as follows: The bed is prepared by spreading over it a sheet of rubber cloth, and over this a blanket. Hot blankets are then wrung out of water of a temperature about as hot as the hand can bear, and with these the patient is enveloped. Two or three thicknesses of dry blankets are superimposed, and the whole retained in position till the patient is free from pain—a time varying from two to six hours. When the pack is applied the patient sweats very profusely, as might be supposed, and after its removal no danger has been found to occur. If the pain is confined to one joint, that joint is enveloped by the hot blanket, which is removed as soon as the pain disappears. One case of chronic rheumatism, extending over a period of seven months, was treated by means of local packings, and after slight relapses, occurring at intervals for five weeks, was discharged perfectly cured. This method presents the advantage in private practice of not shocking the friends of the patient as much as the ice and ice-water packings.—*New York Medical Journal.*

*Curious Incompatibility.*—Chlorate of potassium and iodide of potassium are both entirely harmless in suitable doses. Furthermore, these two salts do not react upon each other in solution, even at a boiling heat. Yet it has been proved that when they are administered together, they do combine in the stomach, producing iodate of potassium, which is poisonous. M. Melsens found that dogs could take the chlorate or iodide in doses of five to seven grammes with impunity, but that a mixture of the two killed them in a few days, with the symptoms of poisoning by iodate of potassium. This combination must, therefore, be avoided. Indeed, as a general rule, the chlorate is so unstable, and so ready to give up its oxygen, that it cannot safely be combined with any substance capable of oxidation.—*American Jour. of Pharmacy.*

*Writing and Reading.*—Let our readers in the country note well the following extract and be encouraged to write for the *Record.* If you write anything original or useful, it will assuredly find its way to all sections, both home and foreign :

" In looking over our foreign exchanges, which include every language of the civilized world, it is a pleasure to see that American medical journalism is constantly growing in the appreciation of foreign professional men. Many of the articles contributed to this periodical within the last year have reappeared in French, German, Italian and Spanish costume. The country doctor, who, from his rural home and limited practice, contributes anything new, which is the fruit of a ripened experience, to the columns of the *Medical and Surgical Reporter*, may rest assured that in less than six months it will have been republished in from fifteen to twenty scientific journals, and in every cultivated language."

*Use of Cold in Fever.*—H. C. Wood, M.D., *American Prac.*, April 1, 1876, remarks: "The use of cold in fever has got beyond the stage of surmise amd speculation ; its value is a demonstrated fact. It is said that one of the most prominent medical teachers in the country recently affirmed that the plan could not be used in private practice on account of the prejudice of patients. Having so employed it, I know that it can be used in private practice, and that the prejudice of patients can be overcome. He who neglects the plan to day is almost as culpable as the surgeon who, shortly after the appearance of the works of Ambrose Pare, refused to use the ligature. Just as certainly as unarrested hemorrhage will kill, just so certainly will neglected fever cases die in which the cold bath would have saved life. Cold will not cure typhoid fever, or *always* avert death, but it will, in many instances, save the patient."

*New Cautery.*—A new cautery seems to attract much attention in Paris. It is the invention of Dr. Paquelin. The accounts of it which have appeared do not give the details of its construction, but it appears to resemble in some particulars the gas cautery employed some years ago in London by the late Mr. A. Bruce. The principle of its construction is, that platinum, or a similar metal, heated to a certain point, becomes instantly incandescent in contact with a gaseous mixture of air and certain hydrocarbon vapors; and that this incandescence is maintained as long as the platinum and gas are in contact. With two hundred grammes of liquid, five hours' work can be done. Any temperature can be maintained steadily, from a dull-red to a brilliant-white heat, and can be instantly varied. Organic liquids, even cold water, do not, it is said, interfere with its activity.—*The Lancet.*

*Select Formulæ—Cincho-Quinine, Strychnia and Iron.*—The following elegant combination of these valuable remedies is the prescription of Dr. J. H. Stephenson, of Leesville, Ohio, who has found it to produce the most favorable results. Take of

| | |
|---|---|
| Cincho-quinine | 64 grs. |
| Strychnia | 4 grs. |
| Chloride iron tincture | 18 fl. drs. |
| Syrup | q. s. |

Triturate the cincho-quinine and strychnia in a glass mortar, adding the tincture of iron gradually and a few drops of nitric acid if necessary, until they are dissolved; filter, and add syrup to make the finished preparation measure one pint.

*Camphorated Tincture of Opium.*—To prepare this, substituting tincture of opium for powdered opium, take of

| | |
|---|---|
| Alcohol | 15 fl. ozs. 96 m. |
| Water | 15 fl. ozs. 96 m. |
| Opium tincture | 12 fl. drs. 48 m. |
| Benzoic acid | 60 grs. |
| Camphor | 40 grs. |
| Anise oil | 1 fl. dr. |
| Honey | 2 ozs. |

Dissolve the benzoic acid, camphor and anise oil in the alchohol, and the honey in the water. Mix the solutions, add the opium tincture, and filter.—*Boston Journal of Chemistry.*

*New Remedy for Burns—Salicylic Acid.*—The method of using it is to form an emulsion with olive oil. This mixture is painted over the ulcerated surface once or twice a day. It gives rise to a slight smarting sensation when first applied, but that soon passes off.—*American Journal of Dental Science*, Feb., 1876.

*Rules in Administering Arsenic.*—In the *Medical Press and Circular*, Dr. H. Griffith gives the following rules for the administration of arsenic:

1. It should never be given where there is a feverish state of the system; a quick pulse and a hot skin contra-indicate its employment.

2. It should never be given shortly after meals—never on an empty stomach.

3. It should not be given in the solid form, nor should it be given in increasing doses. As a rule, five minims of Fowler's solution should be the maximum dose for an adult.

4. The dose should be diminished, or the administration altogether ceased, on the occurrence of pain in the epigastrium, nausea, or irritation of the eyelids.—*Drug. Circular.*

*Rapid Dilatation of Female Urethra.*—By Geo. Jewett, M.D., of Fitchburg, Mass.—Abstract: A girl of fifteen had introduced a crochet needle through urethra into bladder, four days previously; two physicians had failed to remove it; symptoms, those of stone in the bladder. A digital examination verified her statement; the sound also detected foreign body in bladder fixed at both extremities. A number sixteen male sound was passed, followed by passage of index finger, a rupture of the meatus ensued, the finger then passing easily into the bladder. There was some hemorrhage, which ceased spontaneously. The foreign body removed successtully. Patient recovered without incontinency of urine, and in a week was well.—*Condensed from Boston Medical and Surgical Journal*, January 27th, 1876.

*Therapeutic Action of Chlorate of Potash.*—In one section of a long communication on this subject, which is not yet concluded, M. Isambert demonstrates very satisfactorily that chlorate of potash does not undergo any change in passing through the body, neither losing oxygen nor chlorine, but being eliminated en masse by the secretions. This view is supported by the observations and experiments of MM. Laborde, Milton and Gambarini, but it is opposed on theoretical grounds by MM. Berthelot and Gubler, who believe that, like the iodides, it undergoes decomposition.—*Gaz. Med. de Paris.*

*Chloral.*—Dr. Keen says: "I have tried it as a disinfectant in cases of offensive ulcers, abscesses, cancer of the womb, caries, ozena, etc., and I like it better than any other I have tried. It has the advantage over carbolic acid of having very little smell, and over the permanganate that it does not stain the bedding, clothing, etc. As a dressing to ulcers it acts as a stimulant to the granulations, destroys fetor, diminishes the discharge and makes it healthy, and so will often change an unhealthy into a healthy sore. For both these purposes I use it generally about gr. v–xv ad fl. oz. j.

"In erysipelas I have tried it twice as a dressing, and it succeeded well. As an injection into sinuses in strumous abscess, in caries, and other similar cases, the Drs. Dickson write of it in high terms, and I can confirm their observations from my own experience after numerous trials. They also have tried it as an ointment (dr. ss. ad fl. oz. j) with success, and as an injection in gonorrhœa (gr. xx ad. fl. oz. j.,) when the discharge has generally ceased after three days or thereabouts."

*Effect of Extreme Cold on Mind and Body.*—M. Payer, the eminent Arctic explorer, referring to a certain day on which the thermometer indicated 58 degrees Fah. below zero, says that so great an amount of cold paralyzes the will, and that, under its influence, men, from the unsteadiness of their gait, their stammering talk, and the slowness of their mental operations, seem as if they were intoxicated. Another effect of such cold, mentioned by M. Payer, is a tormenting thirst, which is due to the evaporation of the moisture of the body. It is un wholesome, too, to use snow to quench the thirst, as it brings on inflammation of the throat, palate and tongue; besides a temperature of 35½ degrees to 58 degrees below zero, Fah., makes it taste like molten metal. Snow-eaters in the North are considered as feeble and effeminate, in the same way as is an opium-eater in the East.—*Med. Record.*

*Chloroform in Dyspepsia.*—In that form of dyspepsia attended with rapid fermentation of food and evolution of gas soon after a meal, the author of this paper says he finds no remedy to give so prompt and satisfactory relief as chloroform, in doses of fifteen to twenty drops in one or two drachms of simple syrup The gases are expelled from the stomach in a few minutes after swallowing the dose, fermentation is arrested promptly, and very seldom is any unpleasant effect experienced from the chloroform. Some patients speak of a slight intoxicating effect, that passes off in fifteen to twenty minutes. He has never seen it produce drowsiness in these doses, though, if repeated two or three times within an hour, it has a soporific effect upon subjects of mania-a-potu.—*The Medical Brief*, March, 1876.

*Grindelia Robusta—Its Medical Value.*—Dr. H. M. Fisk (*Pacific Medical Journal*, August, 1875,) says that in all cases of iritis he has obtained the happiest results from the use of Grindelia Robusta. It matters not what the cause of the iritis may be—whether gout, rheumatism, scrofula or violence. He gives internally a teaspoonful of the fluid extract every four hours, and externally to the diseased eye a solution of one part to four of water. In asthma he has found its administration of considerable benefit. It controls the distressing nausea and retching attendant upon all stages of cancer of the stomach better than any other remedy he was able to find.

*Enuresis in Childhood.*—At a recent session of the Verein der Aertze in Niederosterreich (*Weiner Med. Presse*), Ultzman delivered an address on incontinence of urine in childhood. Supported by his experience with numerous striking cases which he had treated in his poliklinik, and whose histories he gave at considerable length, he recommended electricity (the faradic current) as the best treatment for the great majority of cases. Recovery occurs in most, and when this cannot be brought about, there is at least a material improvement.—*Exchange.*

*Liquid Glue.*—One part of phosphoric acid, specific gravity 1.120, diluted with 2 parts of water, is nearly neutralized with ammonium carbonate, one part of water added, and then, in a porcelain vessel, sufficient glue dissolved in the liquid to obtain a syrupy consistence. It must be kept in well-closed bottles. The addition of glycerin or sugar would cause the glue to gelatinize.—*Chem. Centralbl.*

*Pruritus.*—The authority of this short notice states that recently he had a most obstinate and severe case of pruritus of the vulva, clitoris, and mons veneris of a fat woman more than sixty years old. So severe was it, that she would cry from the distress. It so overcame her, every way, that she was nearly helpless. After trying a great number of remedies, both internally and externally, for several months, with very little relief, at length, with only the external application of the essence of peppermint to the itching parts, she was almost instantly relieved, with an occasional use of the same.—*Med. and Surg. Reporter*, March, 1876.

*Stoughton Bitters.*—Gentian root, bruised, 4 ounces; orange peel, bruised, 5 ounces; ginger, bruised, and cassia bark, of each, ½ ounce; cardamom seeds, bruised, ¼ ounce; diluted alcohol, 1 gallon; syrup, 2 pints; water, 6 pints; cudbear, or tincture of the same, sufficient. Digest the drugs with the diluted alcohol for a week, then decant the clear liquor, press the residue, and pour on the six pints of water; again macerate for three days, press out the liquid, mix the two tinctures, color to taste, add the syrup, and filter.—*Virginia Medical Monthly.*

*Prevention of Bed-Sores by Sheet Zinc.*—Dr. G. M. Burke, of New Castle, Indiana, states that in cases of protracted illness, the patient being confined to one position, and the parts from pressure become red and inflamed, the threatened ulceration may be prevented by the use of sheet zinc. The zinc should be carefully moulded to the part subjected to pressure, twice in the twenty-four hours being removed and thoroughly washed with cold water.—*American Practitioner.*

*Death from Septic Inoculation.*—The *London Lancet* (March 4) mentions a case of a woman, "a layer-out," in the Isle of Man, who died as the result of inoculation. She was called to lay out the body of a person who had died of gangrenous erysipelas of the arm. The woman, having pricked her finger with a thorn, hesitated, but ultimately undertook the duty. Subsequently she showed symptoms of blood-poisoning, which ended in her death—*Medical Record.*

*Inflamed Testicle.*—Dr. J. Mulveany, in *Medical and Surgical Reporter*, thus treats this affection : The enlarged and very painful testicle to be fomented every hour with hot water, and between times a bag of hot, dry bran to be placed over the scrotum, and to take the following prescription :—

R. Antim. potass. tart.......................................gr. viij,
Tinct. opii ......................................................dr. ij.
Mist. camphoræ ad.........................................oz. viij. M.

Sig.—A tablespoonful to be taken every one, two or three hours, with a teaspoonful of epsom salts in every second dose, till the bowels acted freely.

On the following day he was free from pain, the orchitis much reduced, and, with the aid of a suspensory bandage, he visited and remained for several hours in his place of business.

*Iodoform Pencils.*—

Iodoform in fine powder................10 grammes.
Powdered gum arabic...................0.5 "
Mucilage.......................................q. s.

Divide into 10 equal cylinders, 4 c. m. long; dry in the air.

These pencils are hard, resisting, and capable of being divided into pieces of any length ; they should be preserved from light. They are used with advantage against superficial ulcerations of the uterus. They are introduced into the cavity and allowed to remain, being kept *in situ* by a plug of wadding.—*Medical and Surgical Reporter.*

*Falling Hair.*—A correspondent of the *Medical and Surgical Reporter* asks: "What will prevent the falling of hair?" I have used, for the past ten years, in my own case, and prescribed frequently for others, the following with complete satisfaction :

R. Glycerine.
Tincture capsicum.............................aa. ounces ij,
Oil of bergamot.................................drachm j. M.

Or perfume to suit.

This is to be the only dressing for the hair. Wash the head occasionally with soft water and fine soap.

*Suppression of Urine.*—Poultices of digitalis will in almost every instance procure relief.

*Anti-Rheumatic Mixture.*—In use at the Philadelphia Hospital:

R—Potassii nitratis, dr. j.
Vini colchici radicis, f. dr. j.
Spiritus ætheris nitrosi, f. oz. j.
Syrupi guaiaci, f. oz. ij.
Olei gaultheriæ, gtt. vj.
Aquæ, q. s. ad. f. oz. vj.—M.

Signa.—Dose, a tablespoonful every two hours.—*Drug Circular.*

*Puritus Ani and Vulvæ.*—

R. Calomel.......................... 1 part.
Axunge.......................... 5 to 6 parts.

Wash the parts and annoint freely twice a day, and afterward sprinkle with a powder containing same proportions of starch and camphor.

Equal parts of Ungt. Gallarum and Ungt. Hydrag. Fortius is recommended in pruritus ani.

*Uterne Tonic, Nervine and Alterative.*—

R. Fluid Extract Taraxicum,
" " Scutillaria, aa. ozs. ij.
" " Leptandria,
" " Cimcifuga,
" " Leontice Thal.,
" " Diascoria Vil., aa. oz. j. M.

S. A tablespoonful three times a day.

W. T. G.

*For Dysmenorrhœa.*—

R. Cinquo Quinine.......................... dr. j.
Cimcifuga.......................... grs. xviii.
Extract Stramonicum.......................... grs. vi.
Sacch alb.......................... grs. xxiv.

M. Div. in chart No. xxiv.

S. One three times per day ten days before expected time.

*Chafing.*—H. T. Goldborough, M.D., of Easton, Md., recommends the following as an excellent application for chafing:

R. Zinci Carb.
Lycopodium.......................... aa. oz. j.

S. Apply to the affected part.

*Uterine Tonic and Alterative.*—

R. Fluid Extract Taraxicum.
  " " Helonias Dis.......................aa. oz. ij
  " " Leptandria.
  " " Cimcifuga.
  " " Leontice Thal.
  " " Scutellaria Lat...............aa. oz. j. M.

S. A tablespoonful in water three times a day.

W. T. G.

*Marshall Hall's Nerve Tonic.*—

R. Strychninæ........................................gr. j.
  Acidi Acetici........................................m. xx.
  Alcoholis...........................................dr. ij
  Aquæ ...............................................dr. vj. M.

S. Ten drops for a dose three times or more a day.

In epilepsy, and all cases of nervous exhaustion, whether the result of mental harass or sexual excess, this will be found a good remedy.

*Creosote in Diarrhœa.*—

R. Creosote.....................................gtt. 1 to 5.
  Spirits Ammonia Aromat..........gtt. xx to dr. j.
  Aqua Distil.............................oz. j to oz. jss.

If there is much pain add *compound tincture opii.* dr.ss. to dr.j To be taken at one dose for an adult *pro re nata.*

*Simple Amenorrhœa.*—

R. Fluid Extract Leontice Thal.
  " " Cimcifuga.
  " " Virburnum Prunif.........aa. oz. j.
  Syrup Ferri Iodidi..........................oz. ss. M.

S. A tablespoonful three times a day.

W. T. G.

*For Hysteria and Dysmenorrhœa.*—

Fluid Extract Virburnum Prunif.
  " " " Opulus.
  " " Valerian.
  " " Diascoria Vil..... .........aa. oz. j.
Elixir Opii (McMun's).....................dr. ij to dr.iv.: vel.
Ammon Bromidi..............................oz. ss. to oz.j. M.

S. Dessert-spoonful three times a day.

W. T. G.

*A Third Dentition at the Age Seventy-three.*—M. Echerac relates in the *Gazette des Hopitaux* (October 9th) the remarkable case of an old gentleman, aged seventy-three, who, after the manifestations of nervous symptoms for some time, and an abundant salivation, exhibited in his upper jaw, which had long been dismantled of teeth, some fine ones projecting about two millimeters beyond the edge of the gums. They were six in number—four incisors, one canine, and one small molar. These teeth were neither very white nor very strong, but formed an excellent substitute for those lost. Van Helmont relates a precisely similar case occurring at the same age.—*Medical Times and Gazette.*

*Surgical Anæsthesia in Children.*—This is the method of procedure at the Hospital des Enfans Malades: At eight o'clock the sister administers from three to four grammes (according to the age of the child) of chloral, and the child goes to sleep in twenty minutes. At nine o'clock the dentist passes through the ward and pulls the tooth, at times two teeth, and when the child wakes up, after three or four hours, it is minus its tooth, without suffering, or having seen the dentist. Those who know the pain of having a tooth pulled, and the difficulty of doing this in children, will appreciate in chloral a precious agent.—*Bull. Gen. de Therapeutique.*

*A Prcautionary Measure in Cauterizing the Throat.*—(*Memorabilien—Allg. Weiner med. Zeit.*—Mettenheimer speaks of the dangerous symptoms which have followed cauterization of the throat when the stick of solid caustic has broken and fallen into the larynx. To avoid all danger in case of such an accident, he recommends that the stick be covered with gauze whose meshes are not too fine. In this way cauterization will be just as effectual and unaccompanied by danger. All that is required is that the gauze be frequently replaced.—*Clinic.*

*Waterproof Varnish for Paper, &c.*, is obtained by precipitating a solution of tallow or resin soap by aluminum, iron or copper sulphate, and dissolving the precipitate in a liquid hydrocarbon, or in carbon bisulphide.—*Chem. Centralbl.*

*The Making of Doctors.*—Germany, with a population of 42,-000,000, last year graduated six hundred and sixty physicians, rejecting one hundred and eighty applicants. In the same time the United States, with a population of 40,000,000, graduated three thousand physicians, and remorselessly turned them loose upon the community. We rather incline to the notion that in this country the supply is likely soon to exceed the demand.—*Drug Circular.*

*Nestle's Mother's Milk* and (still more, a modification of it) Faust and Schuster's *Gœttingen infant meal*, Dr. M. considers the best substitute for mother's milk. It contains in 100 parts: 11.5I protein substances, 79.61 carbohydrates, 1.80 inorganic salts, 6.73 water (von Uslar and Polstorff). The inorganic salts contain in 100 parts: 31.70 phosphoric acid, 29.78 potash (Freitag).

*A New Mucilage.*—The *Journal de Pharmacie* states that if to a strong solution of gum-arabic, measuring 8½ fluidounces, a solution of 30 grains of sulphate of aluminium dissolved in two-thirds of an ounce of water be added, a very strong mucilage is formed capable of fastening wood together, or of mending porcelain or glass.—*Scientific American.*

*Cement*—A cement capable of being used where resistance to both the action of water and the action of heat is required is composed by mixing glycerin with dry litharge, so as to constitute a tough paste. For uniting the joints of steam pipes, and other similar applications, this preparation is said to be very satisfactory.—*Pharmacal Gazette.*

*A Convenient Local Anodyne.*—Take of elastic collodion, one ounce; hydrochlorate of morphia, fifteen grains. Dissolve the morphia salt in the collodion. Spread, with a camel hair brush, some of this solution on the painful part, and place some oiled silk over the spot. The effect is stated to be most satisfactory. *London Lancet.*

*Teething Infants.*—When no other disease is found than super-excitation, 1½ to 3 grains bromide of potassium in two or three doses will tranquillize the system.

*The Treatment of Erysipelas* by spirits of camphor is highly recommended by Dr. Hayfelder (*Jour. de Medecine et de Chirurgic Pratique*). He administers 2-4 drops every hour. Under this plan of treatment, he has seen the severity of many cases decidedly diminish.—*Allg. Wiener Med. Zeit.*, May 2, 1876.

*Action of Borax as an Autiseptic.*—Dumas and Schnezler state that they have found the borax coagulates the proptoplasm of the cells, and in this way kills lower organisms; it becomes, then, easy to understand how it can act as an antiseptic.—*Ann. d. Ch. et. d. Phys.*

S*alicylic Acid* has beun used with good results in Germany, in the treatment of recent superficial gangrenous sores, the method being to apply a thin layer of powdered salicylic acid on the surface of the sore, covering it then with wadding.—*Popular Science.*

*To Cement Wood and Iron*—Iron may be cemented in wood by dropping in the recess prepared in the latter a small quantity of a strong solution of sal ammoniac. This causes the iron to rust, rendering it very difficult to extract.—*Scientific American.*

*Experiments* lately made in France show that air laden with coal dust is highly explosive. Several cases of explosion in coal mines have been traced to the action of suspended coal dust when no fire-damp was present.—*Popular Science.*

*Legislation Against Quack Medicines.*—A proposal has recently been made in London to compel every vendor of a patent medicine to declare the ingredients of which it is made before offering the same for sale.—*Medical Record.*

*Balard,* the well known Erench chemist, who died on March 31, at the advanced age of 74 years, was Professor at the College of France, and obtained a world-wide reputation as the discoverer of bromide.—*Medical Record.*

*The Use of the Decimal System* of weights and measures has become compulsory in Austria since the commencement of the present year.—*Clinic.*

*Tonsillitis.*—Beef's gall and vinegar applied hot to the neck will arrest the development of quinsy.

